# Diamond Wire Wear and Its Effect on Surface Quality in Cutting of Monocrystalline Silicon

**DOI:** 10.3390/ma18081768

**Published:** 2025-04-12

**Authors:** Jinguang Du, Yu Wu, Zhen Zhang, Yu Zhang, Wenbin He, Junxiao Geng, Liuyang Duan, Wuyi Ming

**Affiliations:** 1 Henan Provincial Key Laboratory of Intelligent Manufacturing of Mechanical Equipment, Zhengzhou University of Light Industry, Zhengzhou 450002, China; dujinguang@zzuli.edu.cn (J.D.);; 2 Henan Province Engineering Technology Research Center of Green Manufacturing and Precision Measurement, Zhengzhou University of Light Industry, Zhengzhou 450002, China; 3 School of Aerospace Engineering, Huazhong University of Science and Technology, Wuhan 430074, China; 4 Guangdong Provincial Key Laboratory of Digital Manufacturing Equipment, Guangdong HUST Industrial Technology Research Institute, Dongguan 523808, China

**Keywords:** monocrystalline silicon, diamond wire saw (DWS), total thickness variation (TTV), surface roughness, abrasive wear

## Abstract

Monocrystalline silicon is widely used in the semiconductor industry. During wafer machining with a diamond wire saw (DWS), a worn diamond wire can affect the slicing quality. To assess the effect of diamond wire wear on wafer machining, in this study, the impact of diamond wire wear on the wafer’s total thickness variation (TTV) and surface quality was examined at a wire velocity of 1.8 m/s and a feed rate of 0.5 mm/min. Through a single-factor experiment, the effects of the wire velocity, feed rate, and workpiece thickness on diamond wire wear were explored. The outcomes demonstrate that the wear rate was higher in the early and late wear periods, and the wafer machining quality was poor in these two periods. During the stable wear period, the machined wafer exhibited high quality, while the wear rate remained stable. Under the condition of machining the same area of the workpiece, with an increase in wire velocity, the wear quantity for the diamond wire was reduced. As the feed rate and workpiece thickness increased, the wear quantity of the diamond wire increased. The diamond wire wear remained roughly constant when the wire velocity and feed rate increased at the same ratio.

## 1. Introduction

Monocrystalline silicon has excellent mechanical strength and high thermal stability, and it is abundant in raw materials in nature. Thus, a great deal of attention has been paid to it. Monocrystalline silicon is the foundation for manufacturing solar cells and integrated circuits, and photovoltaic cells with silicon wafers as substrates account for more than 85% of the global solar market share [1,2]. Monocrystalline silicon photovoltaic cells have a long lifespan and slow decay, and solar photovoltaic technology accelerates the achievement of global carbon neutrality in energy [3,4]. The machining of monocrystalline silicon is one of the processes involved in producing solar cell substrates [5]. Before the adoption of the DWS, the free abrasive wire saw was adopted for machining silicon crystals. The principle of the free abrasive wire saw is that a steel cable drives cutting fluid containing SiC particles for grinding, and it has the shortcomings of large material loss, challenges in recovering the cutting fluid, low production efficiency, and poor wafer surface quality [6,7]. For a fixed abrasive wire saw, the abrasive particles are bonded to the steel wire, which directly drives them for grinding. This has the advantages of better machining quality, lower environmental pollution, and the cutting fluid being more easily recycled [8].

In recent years, as a type of fixed abrasive wire saw, the DWS machining technique has developed rapidly, with excellent prospects for machining monocrystalline silicon, and it has become the mainstream technology for machining monocrystalline silicon [9,10]. Wang et al. [11] adopted a DWS to cut wafers and proposed an ultrasonic cavitation-assisted method which reduces the sawing force. Cheng et al. [12] investigated the impact of the tension and spacing of the diamond wire on the machining accuracy of the workpiece. The findings suggest that the machining accuracy could be improved by raising the tension of the diamond wire, whereas an increase in spacing would diminish the machining accuracy. When machining monocrystalline silicon with a DWS, Sefene et al. [13] used a traditional cooling system and an electrophoretic-assisted reactive cooling system. The results showed that machining monocrystalline silicon with an electrophoretic-assisted cooling system can reduce the machining cost, improve the quality of the wafer, and reduce diamond wire wear. Wang et al. [14] considered the anisotropy of monocrystalline silicon when machining it with a DWS. When the (100), (110), and (111) crystal faces were cut by the diamond wire, the machining quality was improved at a 30° cutting angle.

When a DWS is used to machine silicon wafers, the cost of the diamond wire accounts for 20% of the entire machining cost [15]. Improving the lifespan of the diamond wire and reducing its cost are important challenges. Optimizing the machining parameters can reduce wear and extend the lifespan of the diamond wire [16]. Several researchers have studied the wear of diamond wire. Hsu et al. [17] adopted gray correlation analysis to refine the machining parameters and obtained the optimal combination of parameters during DWS machining. The optimal combination parameters were used to machine the wafer, and this reduced the abrasion of the diamond wire. Meng et al. [18] utilized a DWS to machine workpieces of the same size, and the results showed that with a rise in wire velocity, the wear of the diamond wire declined, while increasing the feed rate led to greater wear. Researchers have used different methods to characterize diamond wire wear, such as using visual measurement systems and scanning electron microscopy to monitor surface wear on the diamond wire [19], using Raman spectroscopy to qualitatively analyze the wear of abrasive particles [20], or alternatively using a micrometer to gauge the decline in the diamond wire’s diameter [21]. Knoblauch et al. [22] adopted a ring-shaped DWS to machine wafers. They tracked and observed four diamond abrasives on the diamond wire and reported phenomena such as nickel-plating layer shedding and deformation, abrasive particle breaking, and the pulling out of abrasive particles. Gao et al. [23] observed the wear of diamond particles and found that local fragmentation mainly occurred in the early wear stage, while overall fragmentation and detachment of the abrasive particle occurred in the late wear stage. Meßner et al. [24] found that diamond wire wear led to a gradual decline in wire tension. Yang et al. [25] found that the abrasive surface may become graphitized during diamond wire machining of Si wafers, and graphitization will reduce machining efficiency.

Continuous wear of the diamond wire will cause constant changes in wafer quality [26], and adjusting the machining parameters over time can improve the machining quality. Some researchers have studied the machining quality of diamond wires at different stages of wear. Kumar et al. [27] utilized a reciprocating multi-wire cutting machine to cut monocrystalline silicon and found that during machining, the surface roughness decreased, the subsurface cracks decreased, and surface ductility removal became more apparent. Takaaki et al. [28] adopted an unused diamond wire to machine monocrystalline silicon and found that the quality of the wafers improved as machining continued. Yin et al. [29] analyzed the effect of diamond wire wear on the material sawing seam width and found that there was a certain difference between the sawing seam width and wire diameter. Wang et al. [30] constructed a cutting force model for machining monocrystalline silicon with a DWS that considered the wear of the diamond wire, and the outcome indicated that as the wear increased, the quality of the monocrystalline silicon wafers improved.

In summary, it is known that the wear of the diamond wire can affect the machining quality of wafers. Most researchers have analyzed the wafer machining quality of a diamond wire at specific wear stages, but they have not compared the machining quality in different wear states in diamond wire machining of monocrystalline silicon. Therefore, based on the evolution of the wear of the saw wire, the abrasive particles after the wear of saw wire were further classified, and the reasons for the formation of abrasive particles with different wear forms and for the detachment of abrasive particles at different wear stages were discussed. Furthermore, although some research has been conducted on the wear of saw wires, few studies have been carried out on the influence of the process parameters on the wear. Exploring the effect of process parameters on the wear of the saw wire is helpful to predict the life of a saw wire and reduce processing costs. In this study, two experimental studies were conducted. Firstly, cutting experiments on monocrystalline silicon were conducted under constant machining parameters. The changes in the wear morphology of a diamond wire were analyzed, while the influences of the diamond wire in different wear states on the machining quality of monocrystalline silicon were examined. Then, different machining parameters were used to cut a constant area of the wafer, and the influence of these parameters on the diamond wire wear was explored.

## 2. Experimental Methods

### 2.1. Experimental Equipment

**Figure 1:**
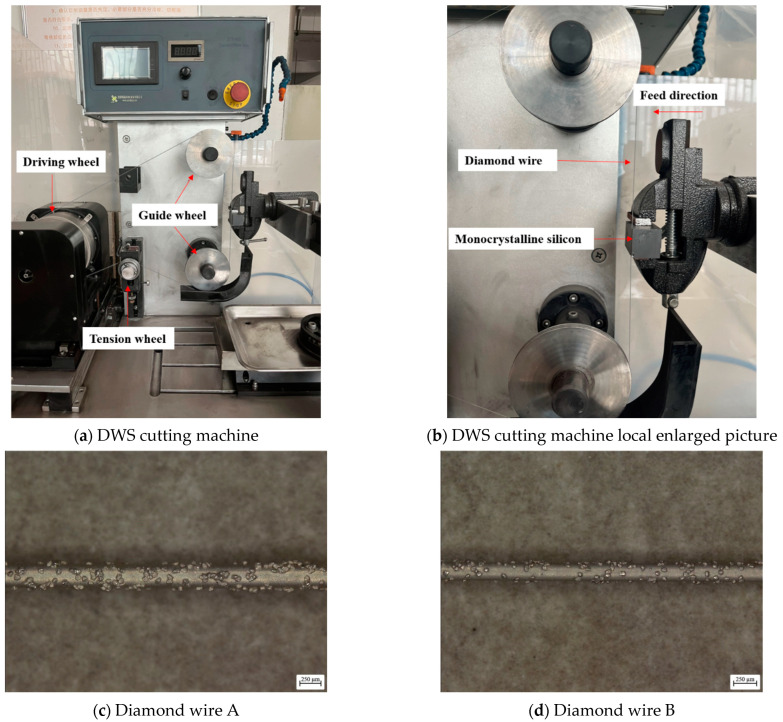
Experimental equipment and diamond wire.

In this study, a reciprocating DWS machine (STX-402, Shenyang Kejing Automation Equipment Co., Ltd., Shenyang, China) was used to cut monocrystalline silicon, as presented in Figure 1a,b. Four workpiece sizes were used, with dimensions of 50 mm × 30 mm × 23 mm, 30 mm × 25 mm × 10 mm, 30 mm × 25 mm × 13 mm, and 30 mm × 25 mm × 16 mm. The driving wheel is responsible for driving the diamond wire to perform reciprocating movement. The two guiding wheels ensure that the diamond wire does not shift, thereby providing slicing accuracy. The tension wheel is responsible for adjusting the tension force during machining, and the CNC system controls the uniform feed of the monocrystalline silicon. Diamond wires of two diameters (Shenyang Kejing Automation Equipment Co., Ltd., Shenyang, China) were used for the cutting experiments. The parameters of the two types of diamond wire are shown in Table 1, and the two types of diamond wire are presented in Figure 1c,d. A 3D super depth-of-field microscopy (DVM6, Leica Microsystems GmbH, Wetzlar, Germany) was adopted to gauge the cutting seam width of the workpiece. The cutting seam width gradually narrows as the machining progresses. Therefore, the position close to the seam root was selected for measurement each time, and the average value of three measured positions was selected. The outside diameter of the diamond wire and the TTV of the wafer were gauged using a micrometer (ES Nscing, Suzhou Suce Testing Equipment Co., Ltd., Suzhou, China). A mark was made on the diamond wire while measuring its diameter. Five points within the marked area were used to calculate the average value, and five locations were used to calculate the TTV. A scanning electron microscope (Phenom XL 100, Thermo Fisher Scientific, Eindhoven, Netherlands) was utilized to examine the silicon wafer and the diamond wire. A portable surface roughness tester (MarSurf PS 10, Mahr GmbH, Göttingen, Germany) was used to gauge the surface roughness. To minimize errors, the roughness was gauged 10 times, and the average value was used.

### 2.2. Design of Experiments

#### 2.2.1. Experimental Design for Diamond Wire Machining of Monocrystalline Silicon with Different Areas

To observe the condition of the diamond wire across various wear stages, cutting experiments on monocrystalline silicon were conducted using diamond wire A from Table 1, and the machining process is illustrated in Table 2. For each group of experiments, a new diamond wire was used. Monocrystalline silicon with dimensions of 50 mm × 30 mm × 23 mm was used. The wire velocity (*V*_s_) and feed rate (*V*_f_) were set to 1.8 m/s and 0.5 mm/min, respectively, and the diamond wire length was 20 m. No cutting fluid was used during the experiment. Although the cutting fluid affects the wear characteristics (such as cooling and lubrication), to expediently analyze the direct interaction between the saw wire and the monocrystalline silicon, the dry cutting condition was used in this study to eliminate the wear mechanism of the saw wire. We paused after each cut and measured the outside diameter of the diamond wire. The feed amount for each cut was 30 mm because the diamond wire produced a wire bow. Therefore, the actual feed was less than 30 mm, which led to different cutting areas during each cut. The 12 cutting seams from Experiment IV were measured, and monocrystalline silicon wafers 2–12 were selected to measure the TTV.

#### 2.2.2. Experimental Design for Diamond Wire Wear

Three factors—wire velocity (*V*_s_), feed rate (*V*_f_), and monocrystalline silicon thickness (*L*)—were used to explore the influence of the machining parameters on diamond wire wear. Diamond wire B in Table 1 was used. The cumulative cutting area of the monocrystalline silicon in each experiment was 650 mm^2^, and a single-factor experiment was carried out as shown in Table 3. The machining parameters were selected based on the adjustable parameter range of the machine tool used and the pre-experiment. In the pre-experiment, we found that when the wire velocity was too low or the feed rate was too high, there was a mismatch between the feed rate and wire velocity, which easily led to the accumulation of wire bow and even wire breakage. Research has shown that during machining, the cutting depth of abrasive particles remains unchanged when the *V*_f_-to-*V*_s_ ratio is constant, even if *V*_s_ and *V*_f_ differ [31]. To explore the influence of the ratio on the wear of the diamond wire, the experiments were designed as shown in Table 4. In each experiment, a cutting area of 650 mm^2^ of monocrystalline silicon was accumulated, and a new diamond wire was used without using cutting fluid. The wire diameter in the marked section was measured before and after the experiment, and the reduction in wire diameter was calculated.

## 3. Results and Discussion

### 3.1. Diamond Wire Wear Analysis

#### 3.1.1. Wear Morphology and Wear Rate of Diamond Wire

(1) Study on the wear of abrasive particles

**Figure 2:**
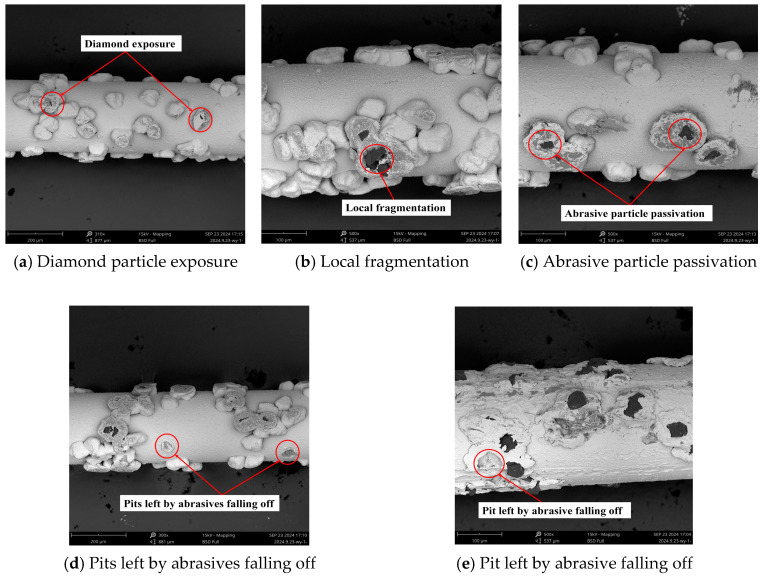
Different wear morphologies of abrasive particles.

**Figure 3:**
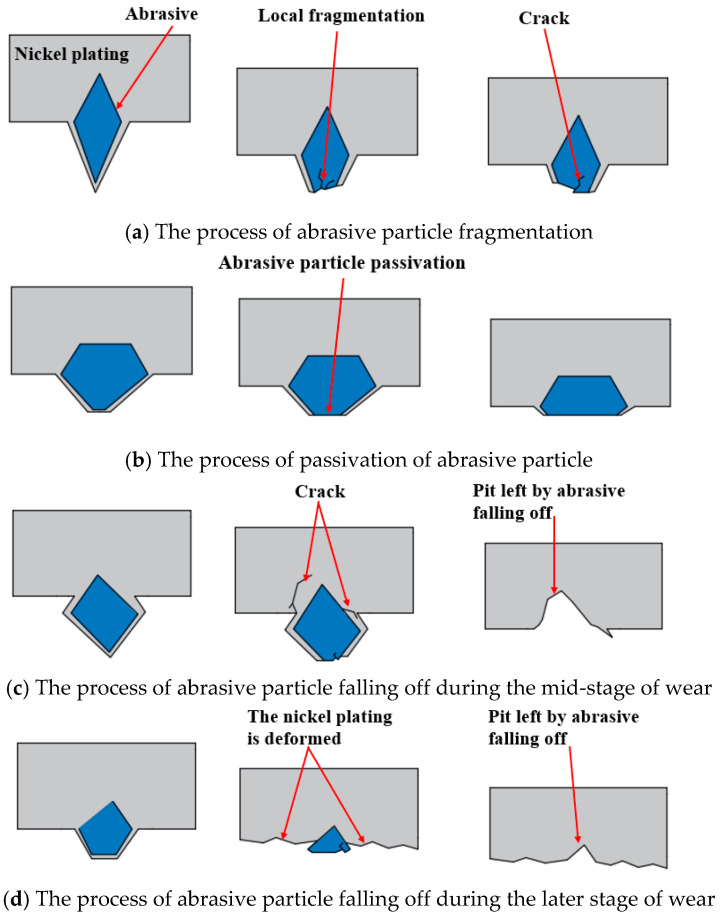
Schematic diagram of the wear process of abrasive particles.

As illustrated in Figure 2, the morphology of abrasive particles after wear may be categorized into four types: diamond particle exposure, local fragmentation, abrasive particle passivation, and detachment of the abrasive particle. As illustrated in Figure 2a, the nickel at the tip of the abrasive particle was removed, and then the diamond particles inside were exposed. The hardness of nickel is relatively less than that of diamond. Once the diamond was uncovered, the wear of the diamond wire slowed down, and the machining ability was improved. This phenomenon primarily occurred in the early period of wear. As illustrated in Figure 2b, the edges of the diamond particles experienced slight breakage due to their inability to withstand mechanical impact and compression, resulting in localized fractures. These broken abrasive particles generated numerous cutting edges, which positively influenced the machining process. Some fine-grained diamonds or diamonds with sharp edges were more prone to local fragmentation. Figure 3a shows the breaking process of abrasive particles, which have large protrusions and sharp edges and are prone to concentrated stress. As the contact stress between the abrasive and the workpiece increased, when the local stress exceeded the fracture strength of the abrasive particles, cracks appeared on the surfaces of the diamond particles, and crack propagation led to local fragmentation. While the fragmented abrasive particles can improve processing efficiency, they also reduce the lifespan of the abrasive. As illustrated in Figure 2c, the abrasive particles were flattened. The passivation of abrasive particles led to the loss of effective cutting edges, causing a decrease in cutting performance. As shown in Figure 3b, for abrasive particles with a large cone angle, the contact area between the abrasive and the workpiece during machining was larger. Compared with abrasive particles with smaller cone angles, the passivated particles had a larger contact area with the workpiece during machining, leading to an increase in surface temperature. In dry cutting conditions, diamond particles may undergo graphitization, which further reduces their cutting ability. Therefore, passivated abrasive particles exhibited weaker cutting performance. As illustrated in Figure 2d,e, pits were present on the diamond wire, accompanied by the detachment of abrasive particles. Figure 2d shows pulling marks on the nickel plating layer at the detachment point, while Figure 2e displays obvious deformation without any pulling phenomenon on the nickel plating layer at the same point. Upon observing the abrasive particles around the detachment point in Figure 2d, it is clear that the surrounding abrasive particles remained relatively intact, indicating that the diamond wire was in a normal wear period. The fact that abrasive particles fell off might be associated with the particle density. When the concentration of abrasive particles per unit area was low, each particle experienced increased mechanical impact, leading to greater stress variation and causing the particles to lose their grip and detach. As shown in Figure 3c, for abrasive particles with insufficient adhesion or those subjected to greater stress, cracks occurred in the nickel plating layer during the cutting process. When the stress exceeded the fracture limit of the nickel plating, the abrasive particles were pulled out along with the nickel plating layer. The nickel plating layer around the detachment point in Figure 2e deformed, and the plating surrounding the diamond particles decreased, which reduced the adhesion between the diamond particles and the nickel layer, ultimately leading to detachment. As shown in Figure 3d, the height of the abrasive protrusion was close to the surface of the saw wire, and the hardness of the nickel plating layer was relatively low, making it more prone to wear. At this point, a large amount of cutting heat was generated during the abrasive machining. Under high-temperature conditions, the nickel plating layer may undergo thermal softening, resulting in deformation of the nickel plating layer.

(2) Wear morphology of diamond wire

**Figure 4:**
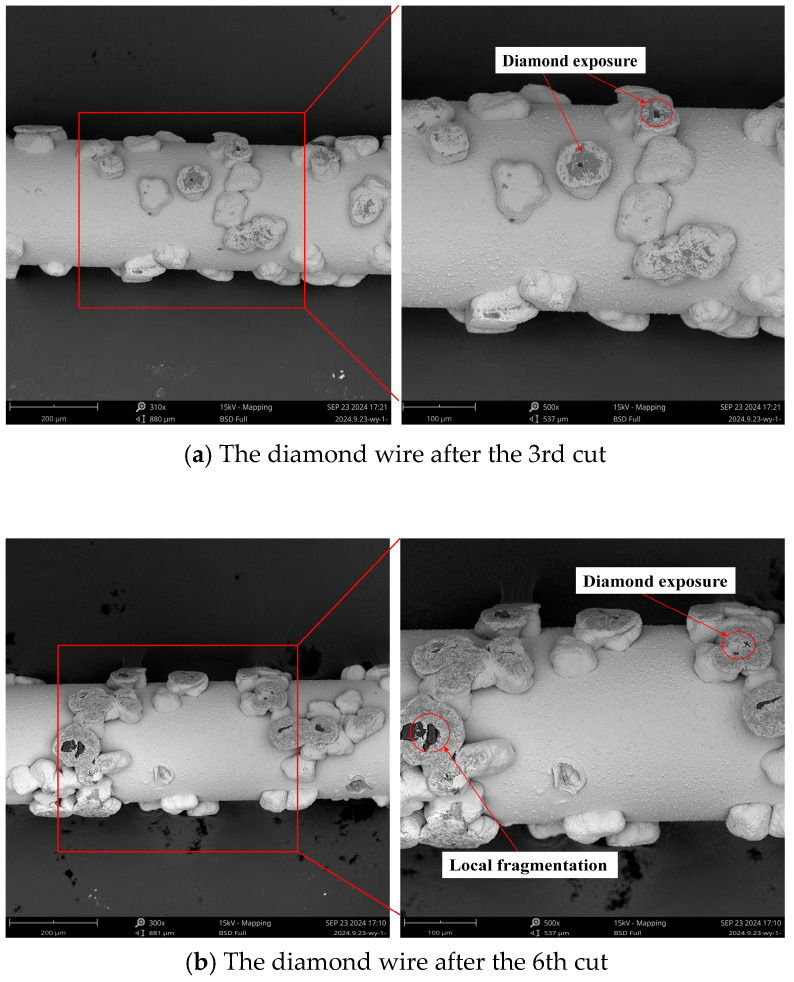

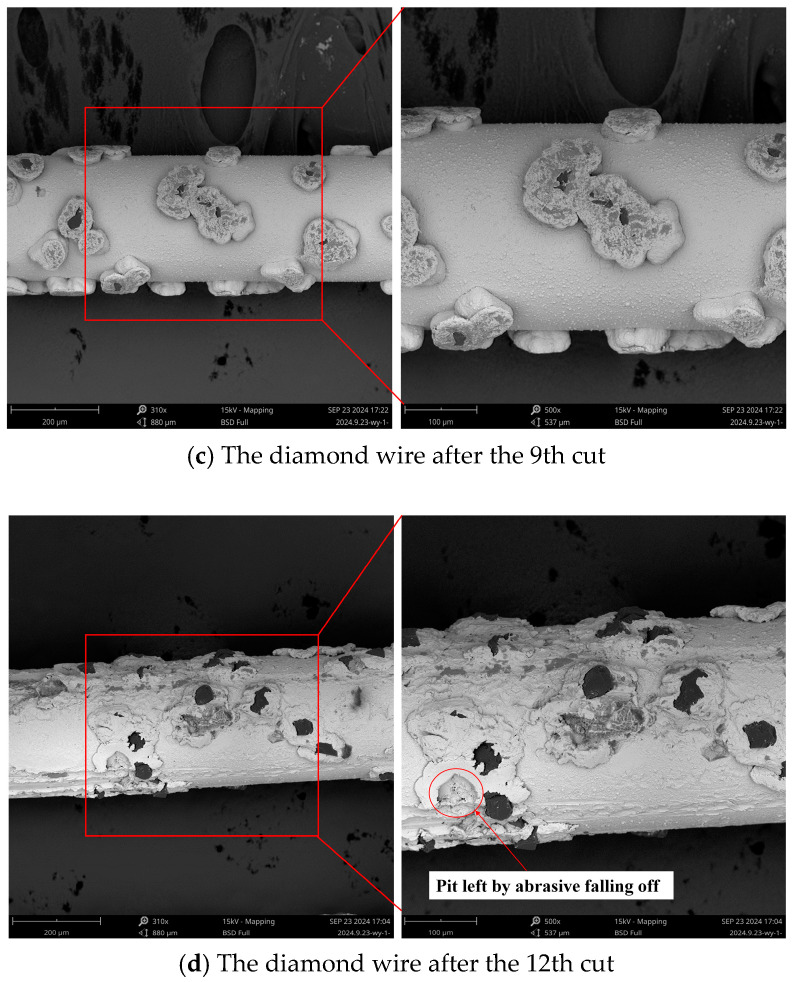
Wear morphology of diamond wire in varying wear states.

**Figure 5:**
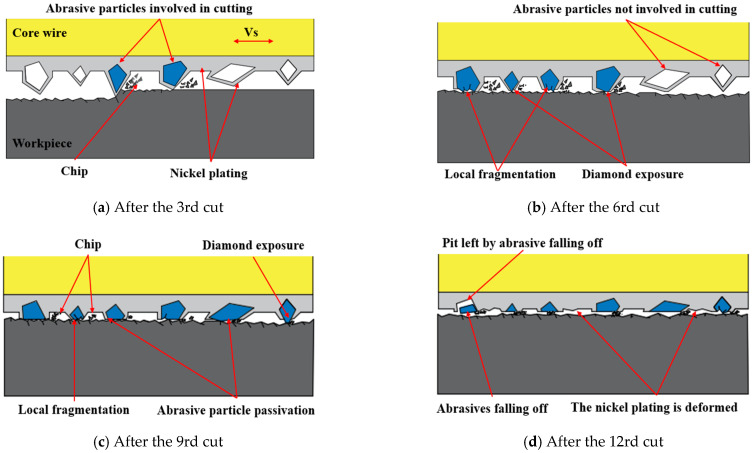
Diamond wire wear evolution diagram.

Figure 4 represents the wear morphology of the diamond wire after the 3rd, 6th, 9th, and 12th cutting of monocrystalline silicon. Figure 4a illustrates an SEM photo of the diamond wire under a cumulative machining area of 1869.9 mm^2^. It can be seen that some abrasive particles exposed their internal diamond, some of them only wore the nickel plating layer, and some of them did not participate in the machining process. As shown in Figure 5a, when the saw wire first participated in machining, the shape of the abrasive particles on the surface varied, and the protruding height was inconsistent. Only the abrasive particles with greater protrusion made contact with the workpiece. Friction occurred first between the nickel plating layer and the workpiece, and once the nickel plating layer was removed, the diamond abrasive particles began to participate in the cutting process. Figure 4b shows an SEM photo of a diamond wire under a cumulative machining area of 3751.3 mm^2^. There were locally fragmented abrasive particles in the diamond wire, and most of the abrasive particles completed the removal of the nickel plating layer. An incredibly small number of abrasive particles remained unworn, and particle detachment was also present. As shown in Figure 5b, as more abrasive particles became involved in cutting, those with higher protrusion and sharper edges broke, causing the average height of the abrasive particles to continuously decrease. Abrasive particles with lower protrusion continued to participate in the cutting process. The breakage occurred because the contact area between the abrasive particles and the workpiece was small, leading to uneven distribution of the cutting force. This caused the cutting force to concentrate in fragile areas, resulting in breakage of the abrasive particles after continuous impact. Figure 4c shows an SEM photo of a diamond wire under a cumulative machining area of 5614.3 mm^2^. It can be seen that some abrasive particles were in a passive state, the tops of the diamond particles became flat, the curvature radius continued to increase, and the protrusion heights of the abrasive particles were comparatively consistent. As shown in Figure 5c, all of the abrasive particles on the saw wire were involved in the cutting, and the contact area between the abrasive particles and the workpiece increased. However, most of the abrasive particles no longer had sharp cutting edges. After continuous wear, the contact area between the abrasive particles and the workpiece gradually increased, and the abrasive particles became blunt. Smaller abrasive particles also broke at this stage. Figure 4d shows an SEM photo of a diamond wire under a cumulative machining area of 7452 mm^2^. It can be seen that the diamond particles fell off, and pits were left. Most of the abrasive particles had low protrusion heights, and the heights continuously approached the diamond wire substrate. The nickel plating layer was deformed and showed signs of detachment. As shown in Figure 5d, most of the abrasive particles were worn down, and the nickel plating layer on the saw wire substrate began to frictionally interact with the workpiece. Due to the decrease in the height of the abrasive protrusion, the contact area between the workpiece and the saw wire increased, generating a large amount of cutting heat. Additionally, the chips and diamond fragments were difficult to discharge, leading to deteriorating machining conditions and a significant reduction in the cutting ability of the saw wire.

**Figure 6:**
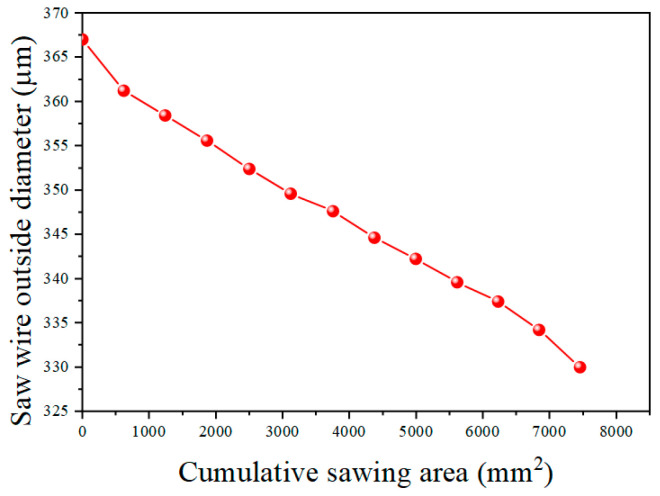
Change in wire diameter with machining area.

**Figure 7:**
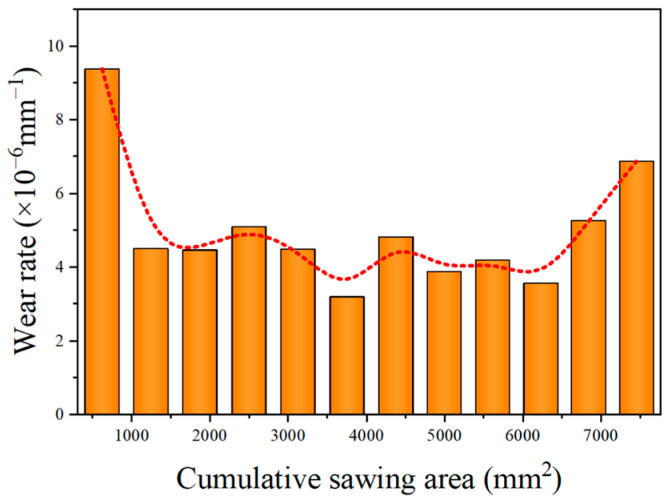
Change in wear rate with machining area.

Figure 6 shows the change in the diamond wire’s diameter with the sawing area. In the course of machining, the diamond wire’s diameter continuously dropped from 367 μm to 330 μm. First, the diameter of the new saw wire was measured, with the initial outer diameter being 367 μm. Then, 12 cutting experiments were conducted, with the workpiece feed set to 30 mm for each cut. However, due to the bowing of the saw wire, the actual cutting length was less than 30 mm. To calculate the total cutting area, the actual cutting area for each cut was determined by measuring the length of the kerf. The cutting areas after each cut were 618.7 mm^2^, 1242 mm^2^, 1869.9 mm^2^, 2497.8 mm^2^, 3123.4 mm^2^, 3751.3 mm^2^, 4374.6 mm^2^, 4993.3 mm^2^, 5614.3 mm^2^, 6230.7 mm^2^, 6840.2 mm^2^, and 7425 mm^2^. After 12 cuts, the outer diameters of the saw wire were 361.2 μm, 358.4 μm, 355.6 μm, 352.4 μm, 349.6 μm, 347.6 μm, 344.6 μm, 342.2 μm, 339.6 μm, 337.4 μm, 334.2 μm, and 330 μm. Since the machining time was the same for each cut, but the machining areas are different, one cannot compare the wear amounts of the diamond wire. Thus, the wear rate is defined as *w*_i_, the diameter of diamond wire at the ith group of the experiment is defined as d_i_, and the cut area of the ith set of experiments is defined as S_i_. The diamond wire wear rate can be expressed using Equation (1) [32]. The correlation between the wire wear and the machining zone is expressed through the wear rate. Figure 7 shows the change in the wear rate with the sawing area:

(1) $$w_{i}=\frac{d_{i+1}-d_{i}}{s_{i+1}-s_{i}}$$

*i* ∈ [0, 12].

During the first cut, the wire wear was the greatest, with the diameter of the saw wire decreasing by 5.8 μm and the wear rate being 9.374 × 10^−6^ mm^−1^. First, the diamond particles were encased in nickel, which is not as hard. Thus, the layer was removed quickly before the diamond particles were exposed. Secondly, the diamond particles on the new diamond wire were exposed at varying heights, with only a limited number of more protruding abrasive particles engaged in cutting. These particles had a higher cutting force per particle, which accelerated their wear. The wear rate sharply decreased during the second and third cuts, indicating that some diamond particles were already exposed. Therefore, the wear rate began to stabilize. The wear rates for the fourth and fifth cuts were 5.096 × 10^−6^ mm^−1^ and 4.476 × 10^−6^ mm^−1^, respectively. After the sixth cut, the diamond wire’s diameter declined by only 2 μm, with a wear rate of 3.185 × 10^−6^ mm^−1^. This was due to the consistent protrusion height of the abrasive particles. Thus, the average cutting force per particle was reduced. The locally fragmented abrasive surface formed new cutting edges, ensuring that its cutting performance remained stable. After the diamond wire finished the ninth machining, the abrasive particles gradually became dull, the tops of the diamond within the nickel plating layer flattened, and no sharp cutting edges remained. The exposure height of the abrasive particles was uniform. Therefore, the wear rate from the seventh to ninth cuts was relatively stable. The interaction area among the abrasive particles and the wafer was relatively large, dispersing the pressure on the diamond wire. The exposure height of the abrasive particles had good consistency, and the load on each abrasive particle was small, resulting in a stable cutting force. After the 12th machining, significant changes in the wear morphology of the wire could be observed; the nickel plating layer underwent deformation and developed scratches. At this point, the nickel plating layer could no longer wrap around the diamond particles, and some diamond particles fell off. The nickel plating layer rubbed against the monocrystalline silicon, causing the diamond wire wear to accelerate. It was observed during the experiment that the wire bow increased while the diamond wire was in operation. From the 10th to the 12th cuts, the wear rate continued to increase, with the wear rate at the 12th cut reaching 6.864 × 10^−6^ mm^−1^. At this stage of wire machining, normal cutting no longer occurred. Due to the decrease in the exposure height of the abrasive particles, there was no chip space between the diamond wire and the wafer, which could lead to three-body wear due to the combined interaction of the diamond wire, chips, and wafer. At this phase, the machining capability of the diamond wire declined rapidly and was severely weakened. The detachment of abrasive particles from the diamond wire mainly occurred at the end of the wire’s lifespan [33]. Continuous cutting may result in wire breakage and a substantial decline in wafer quality. Based on the wear morphology and wear rate, the diamond wire’s lifespan can be divided into distinct wear stages: from the first to third cuts during the initial wear stage; from the fourth to ninth cuts during the stable wear phase; and from the 10th to the 12th cuts during the severe wear phase. The overall trend of the wear rate was consistent with the findings of Zhu et al. [32]. The higher overall wear rate was due to the shorter saw wire length used in our study and the absence of thickened nickel coating on the saw wire.

By observing the different wear states of the diamond wires, it was found that the shape of the abrasive particles varied, and after continuous wear, they developed into different abrasive particle shapes. The variations in shape, size, projection height, and location of the abrasive particles could lead to unpredictable wear conditions. The protruding abrasive particles were the first to participate in cutting and were subjected to a large load, making them more prone to breakage. When the exposed cone angle of the diamond particles was small, it was prone to fracture during friction, leading to the formation of new cutting edges. In contrast, abrasive particles with larger cone angles can withstand greater variable stresses and typically undergo wear and abrasion. The concentration of abrasive particles has a certain impact on its service lifespan. As the diamond wire is a flexible tool, when the density of abrasive particles is low, the mechanical impact on each particle is greater, causing higher variable stress and making the abrasive particles more prone to breakage. If the abrasive density is too low, then this can result in inadequate adhesion of the abrasive particles to the diamond wire, producing their detachment. When the abrasive particle density per unit area was high, the cutting force on each individual particle was relatively low. In this case, the wear on the particles was slower, resulting in a longer service life.

#### 3.1.2. Effect of Machining Parameters on Diamond Wire Wear

**Figure 8:**
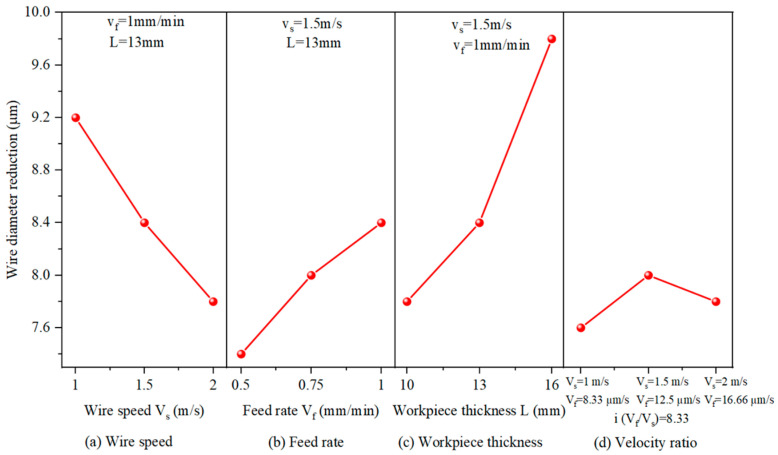
Effect of machining parameters upon the reduction in wire diameter.

Figure 8 illustrates the variation in the reduction in the diamond wire’s diameter after machining the same area under different machining parameters. As illustrated in Figure 8a, with a rising wire velocity, the reduction in the wire diameter decreased. This is because with a rising wire velocity, the quantity of abrasive particles involved in cutting during the same period also increased, causing more abrasive particles to be involved in cutting, which contributed to a decline in the average entry depth of particles into the wafer. The load on each abrasive particle decreased, leading to a more uniform distribution of the cutting force. As a result, the abrasive particle was less likely to break, and the abrasive wear slowed down. However, as the wire velocity increased, there would be an accumulation of heat and chips between the saw wire and the workpiece. The accumulation of heat may lead to an increase in the wafer’s total thickness variation (TTV), while the accumulation of chips can cause damage to the workpiece and degrade the surface quality of the wafer. Additionally, with the increase in wire velocity, the amplitude of the saw wire increased, which is unfavorable for improving the surface quality of the machining. Overall, although increasing the wire velocity reduced the wear of the saw wire, it could have an impact on the machining quality of the wafer.

As illustrated in Figure 8b, with the feed rate’s elevation, the amount of wire wear rose. An elevated feed rate can produce wire bowing. Wire bow increases the tension on the wire, which in turn generates a higher cutting force exerted on the monocrystalline silicon. The load on each abrasive particle increased, intensifying abrasive wear. Simultaneously, the wire’s tension force increased, bringing about more significant elastic deformation, which increased the arc length of interaction linking the diamond wire with the wafer, generating more cutting heat. The temperature in the machining area continuously increased, causing some abrasive particles to undergo graphitization, reducing their hardness, and accelerating wear. As the wire velocity increased, the diamond wire wear decreased. Increasing the feed rate led to greater wear, which is in accordance with the results of Meng et al. [18].

Figure 8c shows the influence of the workpiece thickness on diamond wire abrasion. One can see that as the workpiece thickness rose, the wear accelerated. As the thickness of the silicon increased, the interaction length between the diamond wire and the workpiece extended accordingly, making it difficult for the diamond wire to cool. This may cause some abrasive particles to carbonize, weakening the cutting ability and thereby accelerating diamond wire wear. Simultaneously, as the thickness of the workpiece elevated, the wire bow also increased, which in turn produced a higher cutting force on each abrasive particle. This is not conducive to extending the lifespan of the diamond wire. Thus, the increase in workpiece thickness exacerbated the wear of abrasive particles.

To analyze the effect of the velocity ratio (*V*_f_/*V*_s_) on the wear, as illustrated in Figure 8d, three combinations of machining parameters were selected with a velocity ratio of 8.33. The wire diameter reduction under the three parameter combinations was 7.6 μm, 8.0 μm, and 7.8 μm. The evidence suggests that when machining the same area of monocrystalline silicon cumulatively, the diamond wire wear changes rather little if the wire velocity and feed rate are elevated simultaneously while maintaining an identical ratio. Therefore, increasing the machining parameter proportionally was beneficial for improving machining efficiency without increasing the diamond wire wear.

### 3.2. Effect of Diamond Wire Wear on the Machining Quality of Monocrystalline Silicon

#### 3.2.1. Effect of Diamond Wire Wear on the Total Thickness Variation (TTV) of Wafers

**Figure 9:**
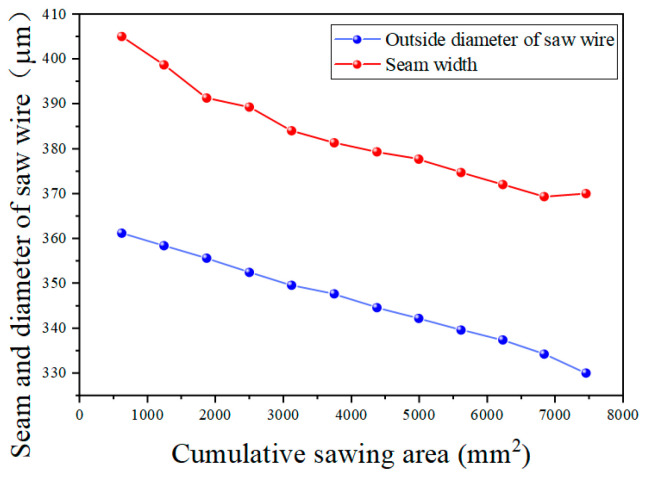
The diameter and cutting seam width of the diamond wire varying with the machining area.

**Figure 10:**
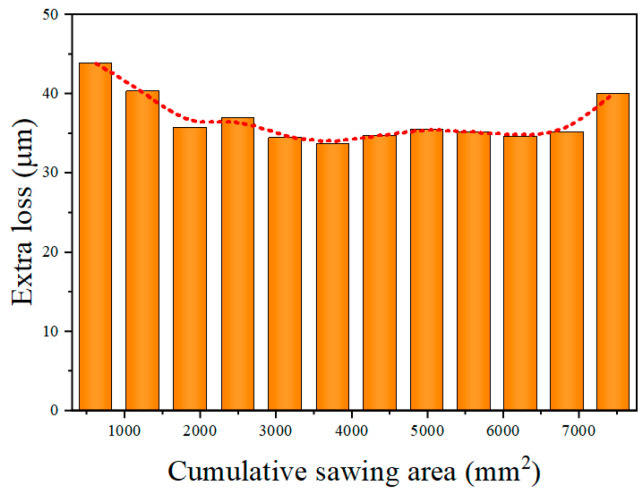
The extra loss varying with the machining area.

**Figure 11:**
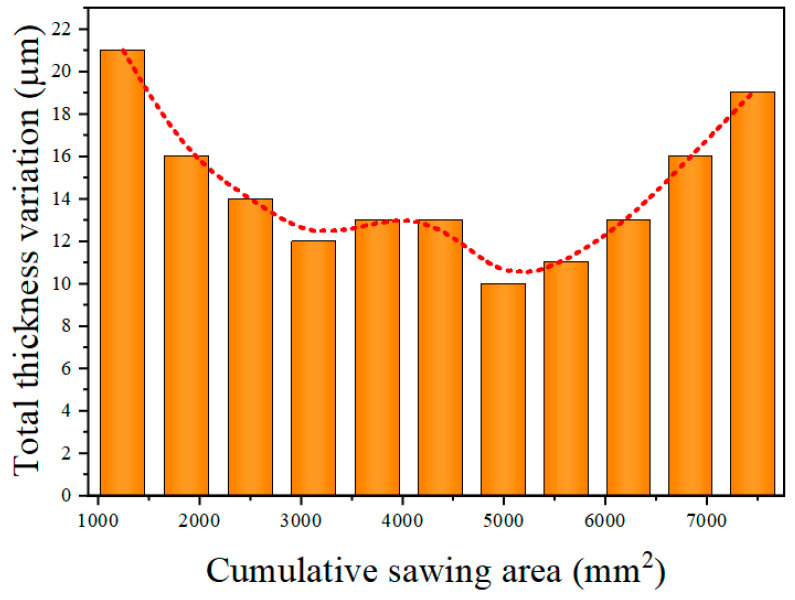
The TTV of the wafer varying with the machining area.

Figure 9 demonstrates the changes in the diamond wire diameter and the cutting seam width. It is evident that the wire diameter consistently declined, while the seam width decreased from the 1st to the 11th cut, with a slight increasing trend observed during the 12th cut. The discrepancy between the seam and the diameter of the wire is referred to as extra loss. Figure 10 shows the variation in extra losses. It can be seen that extra losses decreased successively from the first to third cuts, remained relatively stable from the 4th to 11th cuts and increase during the 12th cut. Figure 11 shows the variation in the TTV of the monocrystalline silicon wafers, which consistently diminished at the beginning of the phase of diamond wire wear and steadily rose at the end of the phase.

As the diamond wire wore continuously, the width of the cutting seam changed, affecting the TTV of the wafer. In the course of machining, the diamond wire wore continuously, leading to a reduction in the cutting seam width. This reduction in seam width increased the TTV of the silicon wafer. Diamond wire wear inevitably led to changes in the cut width. However, when the extra loss stabilized, the TTV of the silicon wafer decreased. From the first to third cuts, the extra loss was 43.8 μm, 40.3 μm, and 35.7 μm, respectively. This suggests that the extra loss was unstable during this period, which caused the large TTV in the early phase of machining. The reason for the instability in the extra loss from the first to third cuts is that as the unused diamond wire started machining, the abrasive particles were uneven in height, creating significant vibrations during its continuous reciprocating motion. Another factor is that the abrasive particles were relatively protruded, and the extent to which the abrasive particles cut into the wafer was large, causing more brittle material removal and increased extra losses. From the 4th to the 10th cuts, the extra loss remained stable at around 35 μm; the extra loss was relatively consistent during this period. This occurred because the abrasive particles on the diamond wire had been sharpened, and the wire’s machining ability gradually stabilized. The projection height of the abrasive particles became uniform, and the shaking of the wire declined. In this procedure, the TTV of the monocrystalline silicon remained relatively small. After the 11th cut, the extra loss began to rise. The feed rate and machining ability were not matched during this period, causing an excessive bow angle, which led to lateral shifting of the wire and changes in the machining trajectory. Another reason is that the abrasive particles became flattened, making it difficult to discharge the chips. A large amount of chips and a few detached diamond particles participated in machining together, leading to an increase in the width of the cutting seam that exceeded the reduction in the diamond wire wear. Overall, this led to a slight increase in the cutting seam width. The machining ability of the diamond wire became unstable in the later stages of wear, causing the TTV of the silicon wafers to continuously increase.

#### 3.2.2. Effect of Diamond Wire Wear on Surface Quality of Wafers

**Figure 12:**
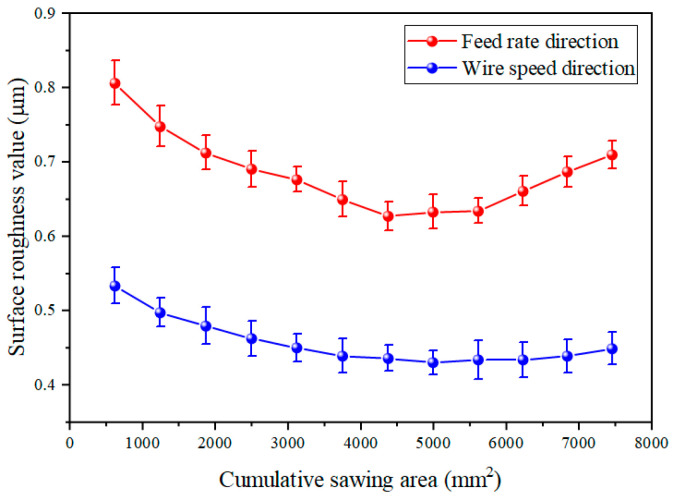
Surface roughness varying with the change in machining area.

Figure 12 illustrates the changes in the surface roughness (Ra) value of the wafer. The Ra value along the direction of the wire velocity decreased continuously from the 1st to 6th cuts, stabilized from the 7th to 10th cuts, and increased during the 11th and 12th cuts. The Ra value along the direction of the feed velocity decreased rapidly from the 1st to 6th cuts, remained stable from the 7th to 9th cuts, and displays an increasing trend from the 9th to 11th cuts. The higher Ra value along the feed velocity direction was due to the silicon moving at a steady velocity during machining. When the wire was reversed, the silicon continued to feed, and the distortion of the diamond wire rose, bringing about an increase in the cutting force. Therefore, saw marks were generated on the wafer [34], which led to a higher roughness value in the feed direction [35].

**Figure 13:**
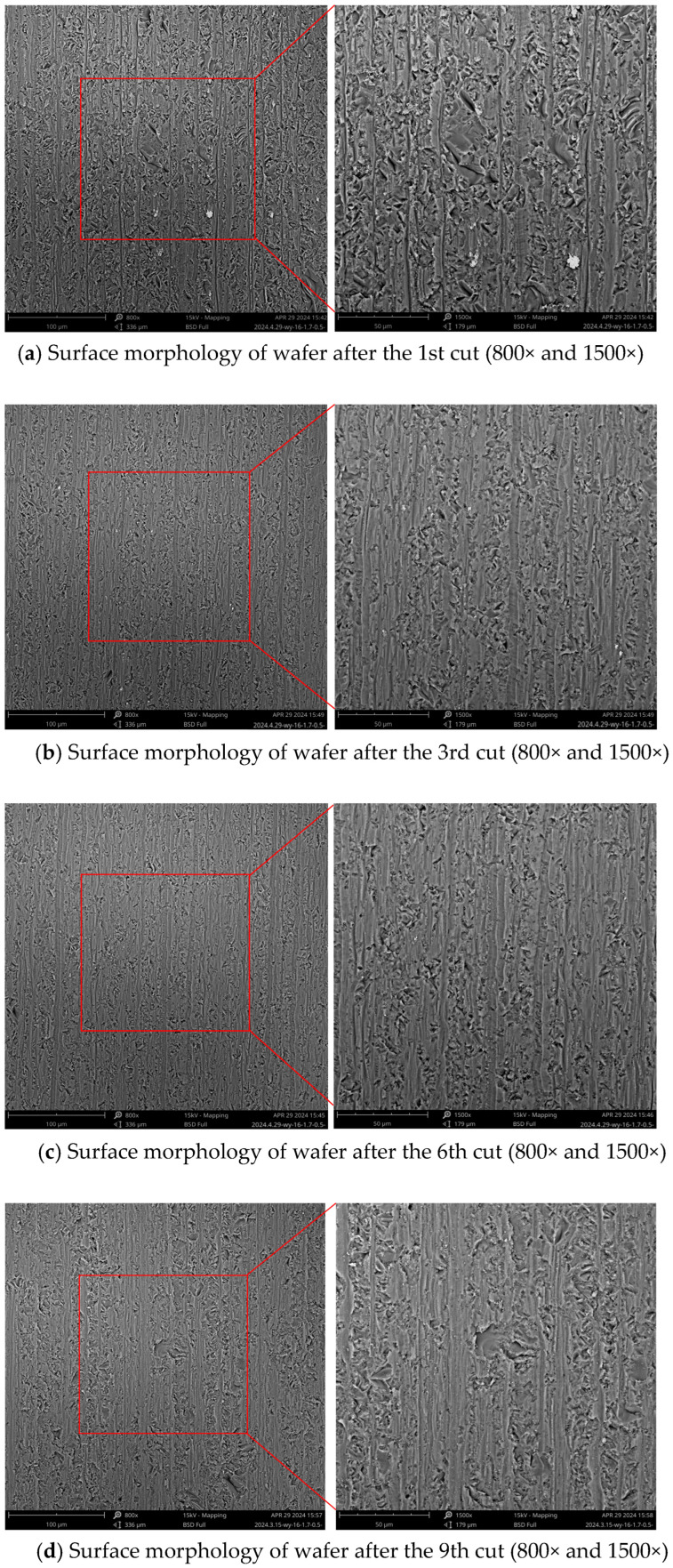

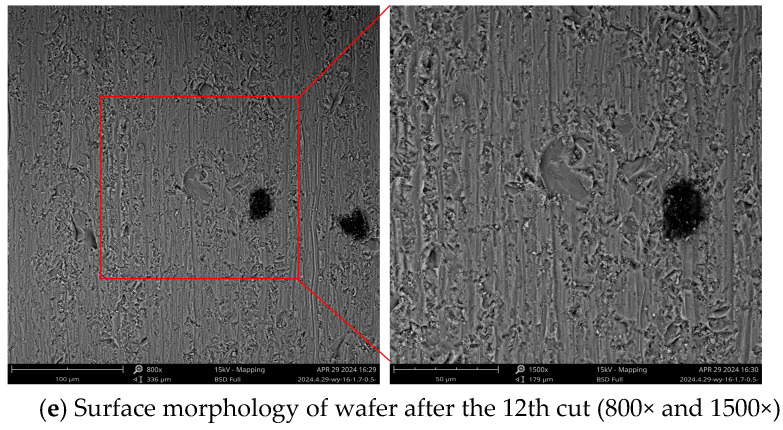
Surface morphology of the wafer changing with the wear of the diamond wire.

**Figure 14:**
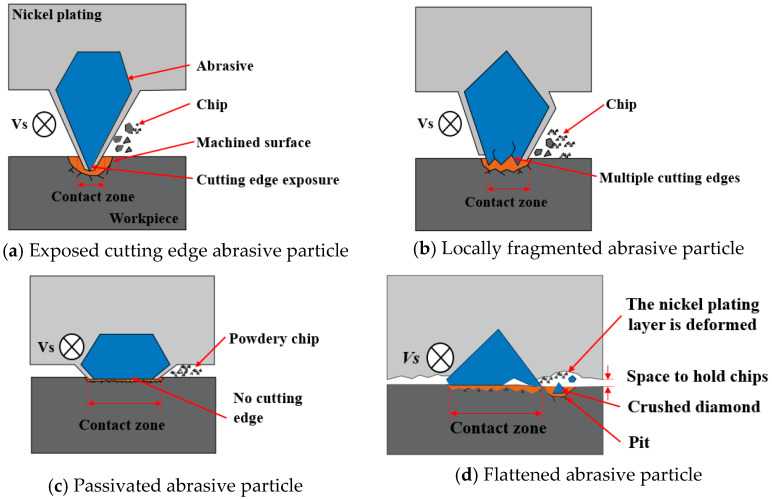
Abrasive machining diagram of different wear types.

**Figure 15:**
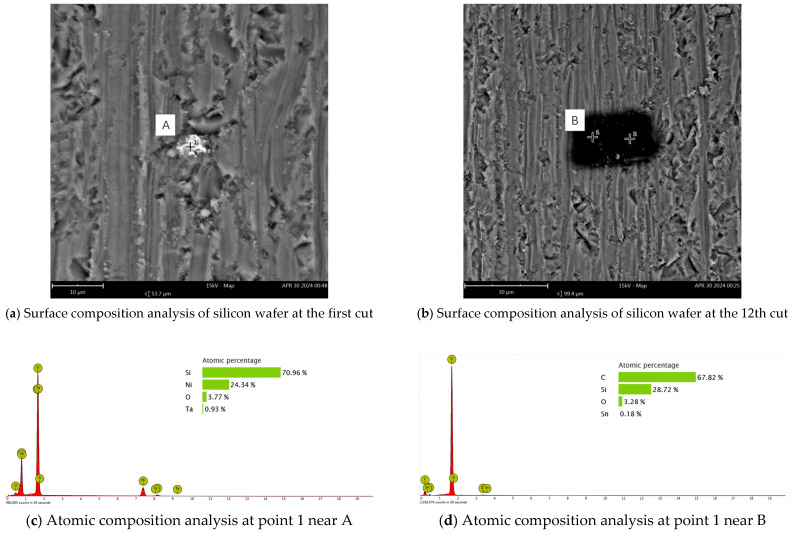
Surface composition analysis of machined silicon wafers.

Figure 13 displays the surface morphology of the silicon after the 1st, 3rd, 6th, 9th, and 12th cutting of the diamond wire. According to Figure 13a, after the total machining area reached 618.7 mm^2^, the surface of the wafer presented significant brittle removal, and the saw marks along the feed path were deeper. The Ra values in the direction of the wire velocity and feed rate were 0.533 μm and 0.806 μm, and the Ra value of the silicon wafer was at its maximum at this time. This is because the projection height of the abrasive particles was uneven. Only abrasive particles with higher protrusions participate in cutting, causing fewer particles to be engaged in the machining, which increases the average cutting force per abrasive particle. This portion of the abrasive particles has a relatively deep cutting depth, leading to deep pits and saw marks, which further facilitates brittle removal of the silicon wafer. This causes significant cutting resistance for the abrasive particles, resulting in a high wear rate. As shown in Figure 14a, at the early stage of cutting, the abrasive particles involved were relatively sharp, and the contact area between the abrasive particles and the workpiece was small. At this stage, the number of abrasive particles engaged in cutting was limited, and the average cutting force per particle was large, resulting in a greater cutting depth. Such abrasive particles left brittle pits on the workpiece surface during cutting. At the beginning of the cutting process, most abrasive particles had a large cutting depth, resulting in a high surface brittleness removal ratio after machining. Based on Figure 15a,c, the surface of the silicon wafer showed white adhesive components. Through atomic composition analysis, in addition to the Si component, the composition of Ni was relatively high, indicating that the white adhesive part was Ni that detached from the abrasive particles. When the new diamond wire started cutting, the nickel plating layer first interacted with the wafer. After the nickel is removed, it may adhere to the surface of the wafer. This phenomenon occurred more frequently in the early wear phase.

In Figure 13b, the morphology of the wafer when the cumulative machining area of the diamond wire reached 1869.9 mm^2^ is displayed. Compared with Figure 13a, it can be observed that the brittle removal was diminished, and the size of the pits decreased. A small amount of Ni still adhered to the wafer. As the cutting area increased from 618.7 mm^2^ to 1869.9 mm^2^, the Ra value declined, indicating that the quality of the silicon wafer was elevated at this phase. According to Figure 4a, the abrasive particles with higher projections continued to wear, and the cone angle at their tops increased continuously, which contributed to the widening of the saw marks. At this stage, some of the abrasive particles completed the removal of the nickel plating layer, while others were continually involved in the machining, leading to a small amount of nickel adhering to the wafer. In comparison with the new diamond wire, more abrasive particles were involved in the machining, reducing the cutting force per particle. As a result, the cutting depth of the abrasive particles became shallower, the brittle removal of silicon wafers decreased, pits became smaller, and the proportion of ductility removal increased. Based on Figure 13a,b, the wear of the diamond wire will influence the quality of the machined wafers.

In Figure 13c, the wafer morphology is displayed for when the cutting area rose from 1869.9 mm^2^ to 3751.3 mm^2^. During this stage, the Ra value in both directions fell progressively. Intuitively, the morphology of the wafer did not exhibit substantial changes. As illustrated in Figure 4b, the wear morphology indicates that the majority of the abrasive particles were engaged in the machining. The abrasive particles with higher protrusions were continuously wearing, while those with lower protrusions were just beginning to participate in the cutting. The abrasive protrusion height across the entire diamond wire was uniform, the average cutting force per abrasive particle was low, and the machining ability of the diamond wire remained stable. As the wire wear increased, the wafer showed increased signs of ductility removal and reduced Ra values, which aligns with the research conducted by Kumar et al. [27]. At this stage, the wire surface mainly contained locally fragmented and passivated abrasive particles. As shown in Figure 14b, the locally fragmented abrasive particles had more cutting edges, and their sharp edges promoted brittleness removal during cutting, thereby improving the material removal rate. The contact area between the passivated particle and the workpiece was large, while the cutting depth was small. This favors the ductile removal of the material surface, but the material removal rate was low.

In Figure 13d, the wafer morphology is displayed with the cutting area of the diamond wire increasing from 3751.3 mm^2^ to 5614.3 mm^2^, and the Ra value in both directions tended to be relatively stable at this stage. The surface of the wafer began to show large pits, indicating that the quality of the wafer began to deteriorate. As illustrated in Figure 4c, most abrasive particles underwent continuous wear, while some became passivated. It can be observed that the saw bow increased during machining because the feed rate remained constant; the cutting ability did not match this feed rate. The change in the saw bow angle increased the cutting force of the abrasive particles on the diamond wire, causing a greater pressing depth for the abrasive particle. At this stage, most of the abrasive particles on the saw wire surface involved in the cutting became dull. As shown in Figure 14c, the contact area between the passivated abrasive particles and the workpiece during cutting was large, resulting in a small cutting depth and reduced surface brittleness removal of the monocrystalline silicon. However, there were more passivated abrasive particles, which would lead to a lower removal rate of material. During machining, the workpiece was fed at a uniform speed. Reduction in the material removal rate led to an increase in the wire bow, which raised the tension force of the saw wire and increased the average cutting depth of the wear particles. Large local cutting forces in some areas of the saw wire led to large pits.

In Figure 13e, the wafer morphology is displayed as the cutting area rose from 5614.3 mm^2^ to 7452 mm^2^, at which point the Ra value in both directions began to increase. Compared with the wafer machined by the diamond wire in the stable wear phase, the ductility removal was reduced while the brittle removal increased. According to Figure 4d, the diamond particles protruding from the diamond wire were relatively low, with some abrasive particles falling off, while the nickel plating layer became deformed. The diamond wire already reached the stage of severe wear, resulting in a significant weakening of the machining ability. During the diamond wire machining, the saw bow continually increased. The length of interaction connecting the diamond wire and the workpiece increased, while the chip space decreased. As shown in Figure 14d, the low protrusion height of the abrasives caused contact between the nickel-plated layer and the workpiece, leading to wear of the nickel-plated layer. Since the hardness of the nickel-plated layer was relatively lower than that of the diamond, the wear rate of the saw wire began to increase. The space between the saw wire and the workpiece was reduced, making it difficult for chips and broken diamonds to be discharged. Diamond debris was sandwiched between the saw wire and the workpiece, and friction occurred, resulting in pits on the surface of the workpiece. At this stage, the saw wire generated significant cutting heat during machining, which may have caused some of the abrasive to carbonize and eventually adhere to the surface of the workpiece. Based on Figure 15b,d, through the atomic composition analysis of the machined wafer, the carbon content was predominant, indicating that the carbonized abrasive particles became embedded in the silicon wafer.

## 4. Conclusions

In this study, cutting tests were performed on monocrystalline silicon, and the surface morphology and wear rate of the diamond wire were analyzed. The machining ability of the diamond wire at different wear stages was analyzed through the TTV and Ra of the wafer. The repercussion of machining parameters on diamond wire wear was explored through single-factor tests. We reached the following conclusions:

(1) The wear rate of the diamond wire was highest at the start of cutting, with the wear rate of the first cut reaching up to 9.374 × 10^−6^ mm^−1^. It remained low during the middle wear stage, where the wear rate of the sixth cut was only 3.185 × 10^−6^ mm^−1^. However, it increased again during the later wear stage, reaching 6.864 × 10^−6^ mm^−1^ at the 12th cut. The morphology of the abrasive particles after wear can be categorized into four types: diamond particle exposure, local fragmentation, abrasive particle passivation, and detachment of the abrasive particle. In addition, abrasive particle detachment not only occurred in the later stages of wear but also during the middle stage of wear when the abrasive particle density on the surface of the saw wire was low or when it was subjected to large force.
(2) In the early stage of diamond wire wear, the TTV of the wafer was relatively large, reaching 21 μm during the second cut. In the middle wear stage of the diamond wire, the TTV was smaller, being only 10 μm during the eighth cut. In the later stage of wear, the TTV began to increase, reaching 19 μm during the 12th cut. Changes in the extra loss affected the TTV of the wafer. During the steady wear state, the fluctuation in extra loss was small, bringing about a low TTV for the silicon wafer.
(3) As the machining area of the diamond wire increased, the Ra value of the monocrystalline silicon wafers displayed a trend of initially declining, followed by an increase. As the wear of the saw wire occurred, the surface roughness value in the wire velocity direction decreased from 0.533 μm to 0.43 μm and then increased to 0.449 μm. In the feed direction, the surface roughness value decreased from 0.806 μm to 0.627 μm and then increased to 0.71 μm. In the early stage of wear, the surface of the wafer exhibited more brittle pits. In the period of stable wear, the silicon wafer showed less brittle removal and more ductility removal, while the surface defects increased in the later wear stage.
(4) When using different machining parameters to cut the same wafer area, the results indicate that diamond wire wear declines with an increase in wire velocity, rises with an increase in the feed rate, and rises with an increase in the cutting thickness of the wafer. When both the feed rate and the wire velocity increased but maintained their ratio, the diamond saw wear remained basically unchanged.

## Figures and Tables

**Table 1:** Diamond wire parameters.

Description	Diamond Wire A	Diamond Wire B
Diameter of wire (μm)	350	250
Average abrasive size (μm)	30–40	20–30
Length of diamond wire (m)	20	20

**Table 2:** Cutting experiment with constant machining parameters.

Experiment	Exp. I	Exp. II	Exp. III	Exp. IV
Wafer (pieces)	3	6	9	12
Cutting time (min)	180	360	540	720

**Table 3:** Single-factor experimental parameters.

Machining Parameter	Exp. I	Exp. II	Exp. III
Wire velocity *V*_s_ (m/s)	1, 1.5, 2	1.5	1.5
Feed rate *V*_f_ (mm/min)	1	0.5, 0.75, 1	1
Workpiece thickness *L* (mm)	13	13	10, 13, 16

**Table 4:** Experimental parameters with the same z ratio (*V*_f_/*V*_s_).

Machining Parameter	Exp. I	Exp. II	Exp. III
Wire velocity *V*_s_ (m/s)	1	1.5	2
Feed rate *V*_f_ (mm/min)	8.33	12.5	16.66
Workpiece thickness *L* (mm)	13	13	13
Velocity ratio (μm/m)	8.33	8.33	8.33

## Data Availability

The original contributions presented in this study are included in the article. Further inquiries can be directed toward the corresponding author.
